# Water Plume Temperature Measurements by an Unmanned Aerial System (UAS)

**DOI:** 10.3390/s17020306

**Published:** 2017-02-07

**Authors:** Anthony DeMario, Pete Lopez, Eli Plewka, Ryan Wix, Hai Xia, Emily Zamora, Dan Gessler, Azer P. Yalin

**Affiliations:** 1 Department of Mechanical Engineering, Colorado State University, Fort Collins, CO 80523, USA; ademario09@gmail.com (A.D.); palopez76@gmail.com (P.L.); eeplewka@gmail.com (E.P.); ryanwix11@gmail.com (R.W.); xia323229@gmail.com (H.X.); emqzamora@gmail.com (E.Z.); azer.yalin@colostate.edu (A.P.Y.); 2 Alden Research Laboratory, Inc. 2000 S. College Ave Suite 300, Fort Collins, CO 80525, USA

**Keywords:** thermal plume, unmanned aerial system, unmanned aerial vehicle, infrared, infrared imaging, temperature profile

## Abstract

We report on the development and testing of a proof of principle water temperature measurement system deployed on an unmanned aerial system (UAS), for field measurements of thermal discharges into water. The primary elements of the system include a quad-copter UAS to which has been integrated, for the first time, both a thermal imaging infrared (IR) camera and an immersible probe that can be dipped below the water surface to obtain vertical water temperature profiles. The IR camera is used to take images of the overall water surface to geo-locate the plume, while the immersible probe provides quantitative temperature depth profiles at specific locations. The full system has been tested including the navigation of the UAS, its ability to safely carry the sensor payload, and the performance of both the IR camera and the temperature probe. Finally, the UAS sensor system was successfully deployed in a pilot field study at a coal burning power plant, and obtained images and temperature profiles of the thermal effluent.

## 1. Introduction

Many industrial processes generate waste heat as a byproduct. A typical method for dissipating the heat is known as once through water-cooling, where water is drawn in from a nearby water source (lake, rive, etc.), passed through a heat exchanger, and discharged back into the water source. This process produces a thermal discharge into the water, creating a thermal plume which can be several degrees Kelvin warmer than the original water temperature. The impact of these plumes on the environment was a factor in the development of regulations for waste heat discharges by the Environmental Protection Agency (EPA). In 1972, the Federal Water Pollution Control Act of 1948 became what is currently referred to as the Clean Water Act (CWA). The CWA revisions made it unlawful to discharge any pollutant from a point source into navigable US waters without a permit [1]. Similar effluent limitations were also placed on thermal discharges from all industrial activities with the goal of protecting aquatic environments.

To address the aforementioned effluent regulations, power plant operators or environmental consultant companies periodically characterize the effluent thermal plumes and examine their impact. Currently, the typical procedure is to pilot small boats, e.g., with two-person crews, through the discharge area. The boats tow thermistors behind them to determine the extent of the plume and establish locations for full-depth temperature readings. At desired locations, full-depth readings are taken by dropping specialized sensors (e.g., Conductivity, Temperature, Depth (CTD) sensors) downward through the water column and recording the data as a function of depth. Data collection with such methods can take a day or more to complete and can be time and labor intensive. More fundamentally, because some of the discharges are near moving water or tidal areas, the plume can change shape, location, and temperature during the measurement period, thereby giving results that may be time dependent. Practical impediments, such as the lack of boat ramps at suitable locations, can also complicate the use of a boat. The use of autonomous boats or submarines may be of interest in the future but these technologies are still under development (e.g., [2]), do not readily allow large field of view imaging (as is useful in this application), and their use would still require shoreline access for launch and recovery of the craft.

The limitations of the existing methods along with the rapid emergence (and regulatory acceptance) of unmanned aerial systems (UAS) are opening the possibility of new measurement paradigms. A UAS water temperature measurement system was conceptually developed by Alden Research Laboratory, Inc. (Fort Collins, CO, USA). Working with a team of Colorado State University mechanical engineering students and their faculty advisor, the concept for field measurements of thermal discharges into water was developed into an operational prototype system. The UAS platforms have the potential to access thermal plumes with temperature sensors without requiring nearby shoreline or boat access. Furthermore, the UAS platforms can be equipped with various imaging modalities, and with global positions systems (GPS), such that data can be geo-referenced. Potential advantages of the UAS approach include the ability to make measurements more quickly (lower cost), with better spatial and/or temporal resolution, with greater reliability (e.g., if a plume can be characterized over a shorter time scale), and in a manner that provides access to more diverse bodies of water and flow types (e.g., those where boat access is very challenging). Where boat access is readily available, the UAS data can augment the understanding of the boat collected data with a surface image of the plume (and additional depth profiles).

The present contribution represents the first simultaneous integration of both an infrared (IR) camera and an immersible temperature probe to a UAS, thereby providing a powerful new capability for measuring and visualizing the temperature of water plumes over relatively large spatial scales (~100 m–kms). Several other groups are developing UAS systems for water resource monitoring (e.g., [3]) but without this simultaneous capability. The use of a UAS to image water surface temperature, but not depth profiles, has been demonstrated by several researchers [4,5,6]; while these approaches are useful, they do not provide the spatial temperature (depth) information needed for the study of thermal effluent streams. One research team has shown the ability to obtain water samples from a UAS platform for subsequent laboratory analysis [7]. Returned samples are useful for chemical or biological analyses but not for in situ temperature determination. The authors are aware of only one other study where an immersible probe has been used to record temperature profiles, which is the recent work of Chung et al. [8]. The system from Chung et al. is oriented towards characterizing temperature fields over smaller spatial scales (~10 m) in lakes or other bodies of water. In contrast to past published efforts, our simultaneous use of both large field of view (FOV) IR imaging and an immersible temperature probe, both integrated to a higher capacity UAS, allows the geo-location of thermal plumes and the collection of temperature profiles over large spatial scales.

The present contribution summarizes the development, validation, and testing of a UAS based temperature imaging system for thermal plumes. The layout of the remainder of the paper is as follows. Section 2 describes the UAS platform and sensor subsystem designs and integration, Section 3 describes the component test results as well as the initial field measurements in the thermal effluent of a power plant, and Section 4 presents the research conclusions.

## 2. UAS Sensor System and Methodology

### 2.1. Overall Design Concept

**Figure 1:**
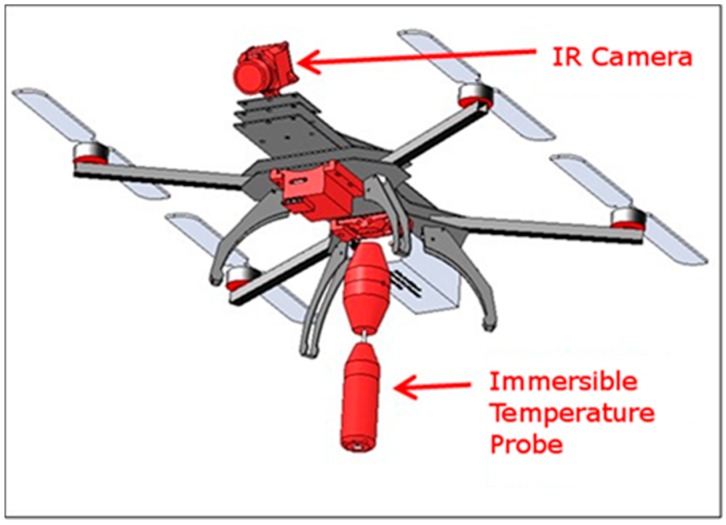
Overall design concept. The UAS flies over the thermal water plume using its navigational (auto-pilot) capabilities. Key components carried by the UAS include an IR camera for thermal imaging of the water surface, an immersible probe that can be lowered and raised to measure the vertical temperature profiles below the water surface, and a controller that stores the data.

The overall design concept is shown in Figure 1 and comprises three integrated functional systems: A quad-copter UAS platform to carry the sensors and camera to custom waypoints with minimal human input, an IR camera for thermal imaging of the water surface to locate the overall plume, and an immersible probe that can be lowered and raised to record the temperature depth profiles below the water surface. The use of an immersible probe, whose depth within the water is controlled by raising and lowering the full UAS system, is found to be a simple and lightweight approach with high versatility. (The use of winch based systems, while possible in principle, tend to be more complex and massive with little benefit in this case.) Ancillary subsystems include a global positioning system (GPS) sensor, data acquisition (DAQ) and storage unit, and mounting and structural elements.

### 2.2. UAS Platform

The system employs the Matrix-I quadcopter from Turbo Ace as a cost-effective aerial platform for proof of concept testing. Favorable characteristics include the ability to safely carry the sensor payload mass (~1.5 kg) with an “all up” (total) mass of about 4.5 kg, thereby allowing the “small UAS” designation by the Federal Aviation Administration (capped at 25 kg (55 pounds)). The Matrix-I has flight parameters consistent with plume mapping requirements, in particular, navigational accuracy better than ±1 m and a range of greater than 300 m. The endurance depends on the payload configuration and choice of batteries. The current system employs a 6 cell, 8000 mAh, 25.2 V LiPo battery with a mass of about 1.1 kg which allows up to 30 min of endurance under optimal flight conditions. Battery sets can be rotated between flights to allow time for drained batteries to be charged while continuing operation with fully charged batteries. The Matrix-I is a four-bladed platform with a carbon fiber frame and high performance propellers (38 cm length) powered by brushless motors. Advanced multi-rotor algorithms offer better performance and reliability than generic hobby quad-copter algorithms and increase motor efficiency. Custom sensor mounting components were 3-D printed from acrylonitrile butadiene styrene (ABS) plastic.

The aircraft can be autonomously controlled with waypoint navigation, including landing and takeoff, via a Pixhawk flight controller. Missions are pre-programmed and the flight status is monitored from the ground using the Mission Planner software. The UAS also includes the ability to use a first-person-view (FPV) system to monitor the flight from the ground station as seen from the UAS. To ensure safe operation, all autopilot flight plans were configured such that the UAS platform never passed directly above people or within 10 m of structures. Additionally, the system was configured to initiate an automated return-to-launch in the event that the drone lost contact to the controller or the battery reached a minimum voltage.

### 2.3. Infrared Camera for Thermal Imaging

An IR camera is mounted on the UAS to perform thermal imaging of the water surface as a means to visualize the overall size and contours of targeted thermal plumes. The use of thermal imaging cameras, based on uncooled (non-cryogenic) IR arrays, for quantitative temperature determination is a relatively mature technology [9] with commercial solutions readily available. The present system employs a FLIR Vue thermal imaging infrared camera specifically designed for UAS use. The camera has a 6.8 mm diameter lens, is lightweight (less than 100 g with the lens), can be powered from the UAS (5 V), and is compatible with multiple camera mounts. Data is transmitted through the First Person View (FPV) live video feed to the ground control station where the video is saved at a frame rate of 7.5 Hz. Image size and field of view are discussed in Section 3.2.

### 2.4. Immersible Temperature Probe

**Figure 2:**
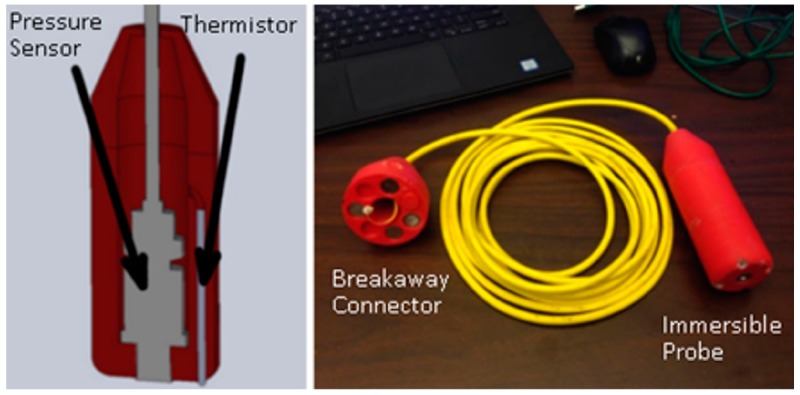
Underwater temperature probe. (**Left**) Probe interior design showing the temperature and pressure (depth) sensors; (**Right**) Umbilical cord with immersible sensor at one end and the breakaway connection at other.

A custom made immersible probe is used to measure temperature depth profiles (relative to the water surface) at desired individual locations to map the thermal plume. The probe can be dipped into the water at multiple locations during a single UAS flight. Temperature measurements are made with a thermistor based probe (Omega HSTH-440000, Omega Engineering, Norwalk, CT, USA) that was selected due to its combination of size, mass, and ease of use in the water environment. To determine the depth of the temperature probe (relative to the water surface), pressure measurements are recorded with a transducer (Honeywell PCB 11, Honeywell, Morris Plains, NJ, USA) and hydrostatic pressure relations are used. A micro-controller (Arduino) samples data from the temperature and pressure sensors at a rate of 2 Hz and the data is stored to a memory card (microSD) for post-flight retrieval. The temperature and pressure sensors were individually tested in dedicated experiments, described in Section 3.3. The thermistor and pressure sensors are contained within a waterproof immersible housing (length ~15 cm and diameter ~3.5 cm), shown in Figure 2, produced with a 3-D printer from ABS plastic. The micro-controller is positioned remotely on the UAS platform and connected to the immersible probe via wires within an umbilical cord (length 6 m). The umbilical cord holds the probe at a fixed position below the UAS such that the probe can sample different water depths as the altitude of the UAS is changed. A safety breakaway connection allowing the lower part of the cord (and immersible probe) to detach from the UAS, in the event that the probe is snagged on the ground or underwater, was developed and flight tested as a safety feature. The mass of the entire probe (including the mounting and umbilical cord) is 900 g.

For safety reasons, the UAS flight controller is set to limit the flight path such that the UAS has a minimum altitude of 2.7 m above the water surface. Given the uncertainty in flight position (controlled versus actual) of ~1 m, this results in limiting the actual UAS altitude to a minimum of ~1.7 m (~5 feet). The corresponding probe immersions can then be to maximum depths in the range of ~3–4 m. This depth limit is sufficient for the desired profiles in this work, but longer cord lengths could be used if needed. Note that despite the uncertainty in controlled UAS flight, the immersive probe measures its position relative to the water surface much more precisely (Section 3.3).

## 3. Results and Discussion

This section contains the results of UAS platform testing, sensor testing, and demonstrative results of the full proof of principle system operating at a power plant.

### 3.1. UAS Flights and Navigation

**Figure 3:**
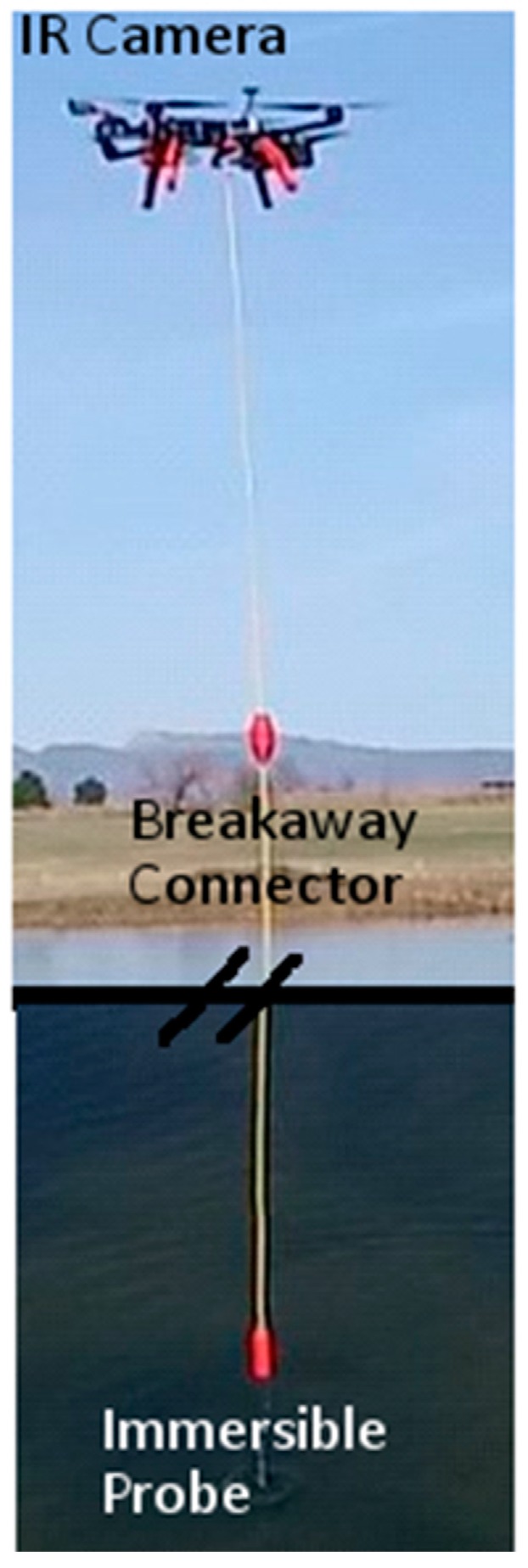
Photograph of the flying UAS in its final flight configuration with all sensors and the umbilical cord. (Full length of the cord is not shown).

The majority of flight testing was done at Christman Field which is an unused air field at Colorado State University in Fort Collins, CO, USA. A series of flight tests were performed examining basic UAS operations and procedures for taking off and landing, flight maneuvers, altitude changes to dip the immersible sensor, and autonomous waypoint navigation. Figure 3 shows a photograph of the flying UAS in its final flight configuration with the sensors and umbilical cord.

Flight testing was used to determine the sensitivity of the UAS to changes in the center of gravity (CG) of the system. The onboard flight controller uses multiple accelerometers for precision flight adjustments. The system performs best when the overall center of gravity coincides with the location of the flight controller. Gusty winds (>~10 m/s for the present system) are an obvious risk for stable flight as they can cause the flight controller to overcompensate pitch-and-roll feedback. The system is particularly vulnerable during landing where a wind gust coinciding with touchdown can trigger an undesired response from the onboard flight controller. Other mounting schemes and/or more sophisticated control schemes can be considered if UAS control becomes problematic due to the change in CG position caused by the umbilical and immersible probe [10]. Flight tests also examined the functionality of the umbilical breakaway mechanism and verified that the mechanism did not inadvertently detach during regular flight operations, but did detach when the immersible probe could not be lifted (as tested by tying it to the ground). The accuracy of the waypoint navigation was examined in a series of flights with different atmospheric conditions. These tests were first conducted over land and then over water. The automated return to the launch system consistently returned the UAS to within 2 m of the launch point.

### 3.2. IR Camera Testing

Infrared camera thermal imaging systems are generally designed for the measurement of solid objects or terrestrial surfaces, not liquid surfaces (i.e., water). Initial testing of the IR camera verified that the camera could capture temperature variations of 1 K (by imaging some known scenes separately measured with thermocouples). The thermal camera was mounted and flown on the UAS to examine the field of view and the effects of the resolution and shutter speed settings. Test flights for the thermal camera were performed by mounting it to the UAS, as well as in separate flights where it was mounted to a fixed wing aircraft (Cessna, Cessna Aircraft Company, Wichita, KS, USA) flying over the discharge reservoir at the Rawhide Energy Station near Wellington, CO, USA. The field of view (on the water surface) attainable by the camera is defined by the flight altitude and angular extent of the image, where the latter is determined by the lens and camera optical design. For typical altitudes of ~120 m, the IR camera yielded image dimensions of ~100 m × 80 m based on the view angles of 44 and 36 degrees, with an image resolution of 333 × 256 pixels. This field of view is generally too small to image a full thermal plume, such that multiple images should be “stitched” (combined) together to achieve the desired larger view. In this work, we have manually combined the images using a commercial software package (Photoshop), though more sophisticate stitching combined with geo-tagged images could be used in the future. Testing confirmed that the camera can be used to provide qualitative images of the thermal plume, allowing the determination of the plume contour. Examples of IR camera images are shown in Section 3.4.

### 3.3. Immersible Temperature Probe Testing

**Figure 4:**
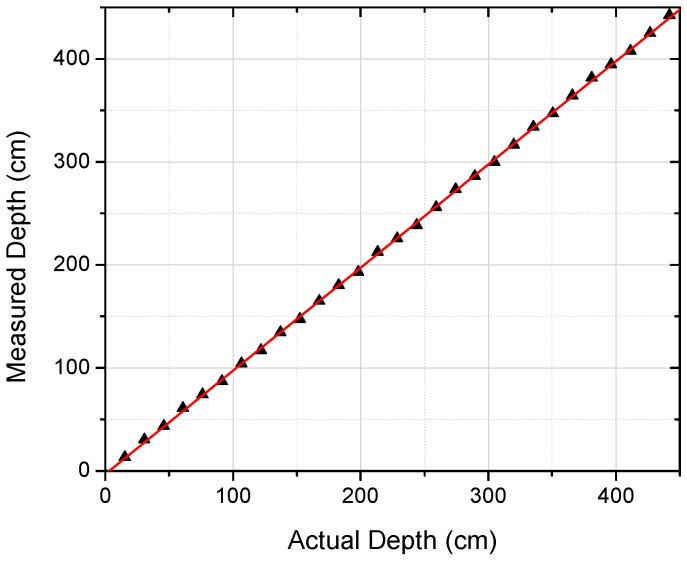
Test results of the depth probe showing the relationship between measured and expected depth.

Tests were performed to confirm the performance of both the temperature and depth measurements from the immersible probe. For the former, temperature readings of a water bath from the immersible probe thermistor were compared against a reference thermocouple and were found to agree to within ±1 K, which is taken as the uncertainty (error bar) for temperature readings. The approach for determining the sensor depth is to measure the local pressure and then infer depth (relative to the water surface) using hydrostatic pressure relations, i.e., pressure increases by ~96 kPa/m of depth in fresh water. The sensor depth readings were calibrated (to 5 m depth) by immersing the sensor to varying known depths within a column of water. As shown in Figure 4, the agreement (between known and measured position) was very linear with a slope close to unity and zero offset (the linear fit yields a slope of 1.002 and an offset of −3.0 cm with *R*^2^ = 0.99993) and showed that depth could be measured to within ±7 cm.

### 3.4. Field Testing of the UAS Thermal Plume Mapping

The full UAS sensor system was tested on 21 April 2016 during three separate flights at the Rawhide Energy Station (RES) in Wellington, CO, USA. The RES has a single 280 MW coal burning unit, four 65 MW single-cycle natural gas turbines and one 128 MW single cycle gas turbine. Testing comprised of flying a survey mission at an altitude of ~120 m and obtaining both IR images as well as subsurface water temperature profiles, by dipping the temperature sensor at critical points in the plume. Winds were relatively strong at ~6 m/s to 9 m/s, and the UAS flew successfully, with the payload, under these conditions.

**Figure 5:**
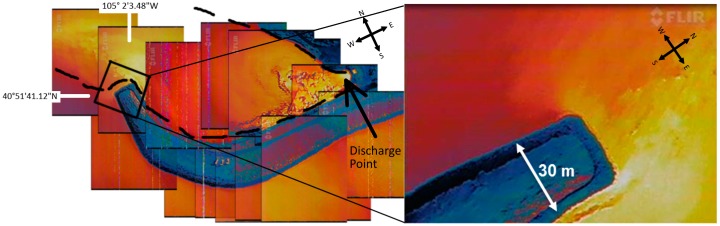
(**Left**) Composite image of the water surface showing thermal discharge from the Rawhide Energy Station obtained by the UAS IR camera. The location of the discharge point is indicated while the approximate plume contour is shown with a dashed line; (**Right**) Single image of a thermal plume at the end of the discharge guide berm.

Imaging of the thermal plume with the IR camera was performed by ascending to a flight ceiling of ~120 m and making several passes over the water to image the extents of the plume. The left panel of Figure 5 shows a composite image of the water discharge area, revealing the thermal plume. The infrared camera used during the flight automatically scales each image based on the range of temperatures in the individual image. Note that other available UAS compatible cameras, for example ICI 9640 P-series (Infrared Cameras, INC., Beaumont, TX, USA), allow consistent color scaling over multiple image acquisitions resulting in more continuous composite images. The right panel of Figure 5 shows the results of a single infrared image.

**Figure 6:**
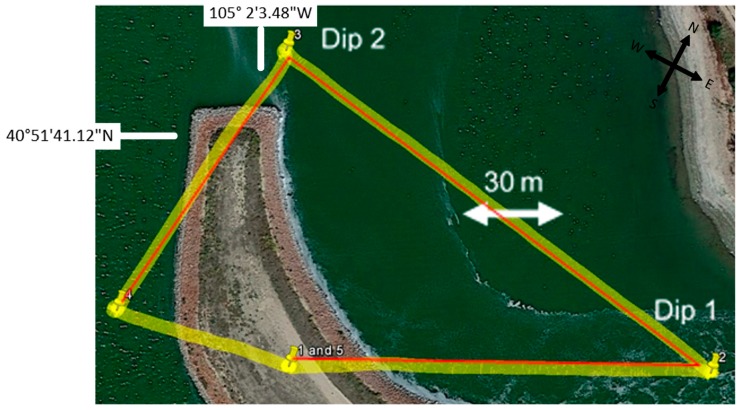
Flight path (actual in red and programmed in yellow) showing the three dip locations for the subsurface water temperature profiles at the Rawhide Energy Station.

The final aspect of UAS flight testing was to conduct three immersion dips to measure the subsurface water temperature profiles. Figure 6 shows a map view of the discharge area indicating the dip locations. The UAS took off from the nearby peninsula and flew approximately 140 m to the first dip location, 190 m to the second dip, 150 m to the third dip, and finally 80 m to return and land at the original launch location. The entire flight was autonomous and executed without input from the pilot.

**Figure 7:**
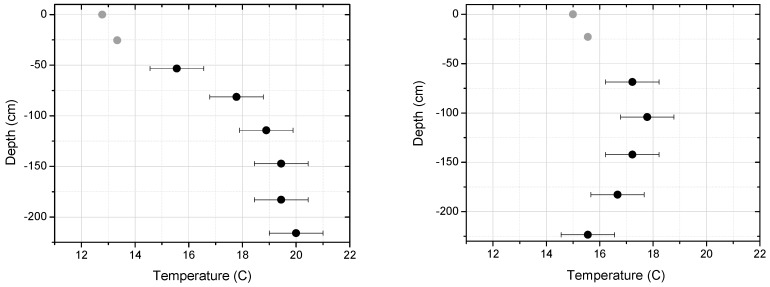
Temperature profiles below the water surface from two dips at the Rawhide Energy Station (**Left**: dip 1, **Right**: dip 2).

Example temperature depth profiles from the immersible UAS probe are shown in Figure 7. The plotted temperature profiles are referenced to the water surface at depth zero. Dip 1 was relatively close to the outflow point while Dip 2 was further away and at the tip of a peninsula. For depths below ~1 m, the Dip 1 profile shows warmer temperatures than Dip 2, consistent with the greater proximity to the discharge source location. The profiles were obtained by lowering the probe through the water at a speed of ~50–70 cm/s (the exact value was influenced by water forces on the probe) with data gathered at a rate of 2 Hz. Each downward dip was ~2.5 m in extent, corresponding to a time extent of ~4–5 s that yielded 8–10 data points with a spacing ~25–30 cm. The top several points (recorded in the first 2 s of immersion) in each dip profile are colored grey to indicate that the thermistor readings may not have sufficiently stabilized for these specific measurements. There is a relatively large temperature jump that occurs at the instant of immersion, due to the sudden jump from cooler air (~13 °C) to warmer water, such that more time is required for stabilization to within the uncertainty (±1 K) for points near the surface where the temperature change is largest. Overall, the system has shown the ability to dip the immersion probe and record temperature profiles. Future work should further examine the probe response time and modifications to the dip speed should be made depending on the data needs; for example, the probe can be lowered more slowly if the data at the top of the water layer is critical.

## 4. Conclusions

The present research has developed a UAS sensor package for the study of thermal plumes in water, particularly for the examination of thermal effluents from power plants. Such approaches can potentially displace (or complement) the boat-based operations that are currently widely used for effluent characterizations for regulatory and environmental assessments. A quad-copter UAS has been modified to carry the sensors. With the appropriate consideration of the center of gravity location, stable flights could still be achieved with the modified configuration. An attractive feature of UAS operation is the ability to navigate via automated waypoints as demonstrated in the present research. The two main required sensors for the thermal characterization are an infrared thermal imaging camera, used for qualitative imaging of thermal gradients to reveal the overall plume location, and an immersible probe to record the temperature depth profiles at different fixed point locations in the plume. Both sensor subsystems were characterized in controlled tests and were then integrated to the UAS. The immersible probe hangs below the UAS, such that it can be dipped in/out of the water by lowering/raising the overall UAS, and is connected to the UAS via an umbilical cord. For safety reasons, the umbilical cord contains a breakaway mechanism allowing the probe (and the lower part of the cord) to disconnect and fall into the water in the event that the probe is snagged or cannot be retracted.

The full system was flight tested in a proof of concept study at the Rawhide Energy Station including waypoint navigation, imaging of the thermal plume from the IR camera, and acquisition of the temperature depth profiles. The IR camera used in the present work provided a proof of concept demonstration but could ultimately be replaced with a more advanced unit allowing more capability for geotagging of the images, higher resolution, on board storage, and the use of automated image stitching software. The dip probe data demonstrates that the water temperature can be measured at the surface and below the surface using a UAS. Testing is required for each UAS system to understand the accuracy and time response of the temperature probe.

The most significant shortcomings of the system are the limited flight duration and weather and regulatory challenges. The current configuration cannot operate in precipitation and is limited to 20–35 min of flight time (depending on flight profile, wind, number of dips, etc.), which can be limiting for sampling large plumes. However, these limitations can be largely overcome by using a larger UAS and continued improvements in battery technology. The fully loaded UAS is currently limited to flying with wind speeds less than ~10 m/s. The wind speed limitation is expected to improve if a larger UAS is used and as flight controller technology improves. The most significant wind limitation is during landing, where a gust of wind can adversely affect flight performance when the UAS is at low power near the ground. More advanced flight controllers would also benefit the performance in high winds and general stability, and one could integrate the sensor control and data storage functions into the flight controller itself (obviating the need for the Arduino currently used for this purpose).

In the United States, the operation of UAS systems (without additional certification) is limited to 122 m (400 feet) above ground level by the Federal Aviation Administration. This limits the area which can be seen in a single image. A wide-angle camera lens or oblique view of the terrain can improve the coverage footprint; however, a wide-angle lens introduces distortion and oblique views are more challenging in terms of determining the position of the plume.
